# Novel Approach to Support Rapid Data Collection, Management, and Visualization During the COVID-19 Outbreak Response in the World Health Organization African Region: Development of a Data Summarization and Visualization Tool

**DOI:** 10.2196/20355

**Published:** 2020-10-14

**Authors:** Kamran Ahmed, Muhammad Arish Bukhari, Tamayi Mlanda, Jean Paul Kimenyi, Polly Wallace, Charles Okot Lukoya, Esther L Hamblion, Benido Impouma

**Affiliations:** 1 Regional Office for Africa World Health Organization Brazzaville Congo; 2 Australian National University Canberra Australia

**Keywords:** COVID-19, health information management, data collection, visualization, EWARS, WHO African region, Go.Data, outbreak, pandemic, health emergencies

## Abstract

**Background:**

The COVID-19 pandemic has created unprecedented challenges to the systematic and timely sharing of COVID-19 field data collection and management. The World Health Organization (WHO) is working with health partners on the rollout and implementation of a robust electronic field data collection platform. The delay in the deployment and rollout of this electronic platform in the WHO African Region, as a consequence of the application of large-scale public health and social measures including movement restrictions and geographical area quarantine, left a gap between data collection and management. This lead to the need to develop interim data management solutions to accurately monitor the evolution of the pandemic and support the deployment of appropriate public health interventions.

**Objective:**

The aim of this study is to review the design, development, and implementation of the COVID-19 Data Summarization and Visualization (DSV) tool as a rapidly deployable solution to fill this critical data collection gap as an interim solution.

**Methods:**

This paper reviews the processes undertaken to research and develop a tool to bridge the data collection gap between the onset of a COVID-19 outbreak and the start of data collection using a prioritized electronic platform such as Go.Data in the WHO African Region.

**Results:**

In anticipation of the implementation of a prioritized tool for field data collection, the DSV tool was deployed in 18 member states for COVID-19 outbreak data management. We highlight preliminary findings and lessons learned from the DSV tool deployment in the WHO African Region.

**Conclusions:**

We developed a rapidly deployable tool for COVID-19 data collection and visualization in the WHO African Region. The lessons drawn on this experience offer an opportunity to learn and apply these to improve future similar public health informatics initiatives in an outbreak or similar humanitarian setting, particularly in low- and middle-income countries.

## Introduction

In December 2019, a cluster of cases emerged in China caused by a novel coronavirus disease, officially named COVID-19 by the World Health Organization (WHO) [1]. Since, COVID-19 has spread widely with a rapid global spread in just a few months [2]. The WHO’s Director-General declared COVID-19 a Public Health Emergency of International Concern on January 30, 2020, and later as a pandemic on March 11, 2020, urging all countries to take urgent and aggressive actions for detecting, tracing, isolating, and treating active cases, and for preventing transmission to reduce COVID-19–related morbidities and mortalities [3]. As of May 19, 2020, there have been around 4.7 million laboratory confirmed cases of COVID-19 reported across the world [4]. This novel coronavirus first arrived on the African continent in February, with the first few cases detected in Egypt and Algeria [5]. As of May 19, 2020, all WHO African Region member states are affected with 47,953 confirmed cases and 1488 reported deaths with a case-fatality ratio of 3.1% [6].

During any disease outbreak or health emergency, timely access to validated data and its translation into evidence to support swift public health actions and decision making is one of the biggest challenges faced by many public health experts, especially in resource-poor settings. This is especially true for settings where routine public health surveillance systems are underperforming or nonexistent, or may be disrupted during the COVID-19 crisis. Such delays in outbreak control during an emergency response results in delayed case detection and public health actions to mitigate onward transmission. Consequently, higher mortalities with higher rates of disease transmission in communities are inevitable. To address such delays, the WHO recommends implementation of a disease early warning system, known as the Early Warning, Alert and Response System (EWARS), within 3-10 days of the onset of an emergency’s acute phase as one of the priority interventions to mitigate the negative health consequences resulting from the acute emergency or humanitarian event [7].

The EWARS system supports any existing health system and facilitates collection of essential, minimal data on prioritized epidemic-prone or selected diseases with significant public health consequences to enable rapid analysis of trends for outbreak or emergency responses in humanitarian settings. Enhancing the EWARS system using robust electronic data systems could be harnessed as a powerful tool by outbreak response teams for collecting vital epidemiological data to support appropriate and timely action during emergencies. The WHO has developed various electronic tools and platforms such as the electronic Disease Early Warning System (eDEWS) and EWARS-in-a-Box to support EWARS surveillance data collection, management, and analysis to inform timely public health actions and evidence-based decision making in humanitarian settings. Furthermore, these electronic systems have been adapted to support the surveillance component of the electronic Integrated Disease Surveillance and Response (eIDSR) and to facilitate early detection of prioritized epidemic-prone diseases [8-10].

COVID-19 is a current focus of surveillance, contact tracing, and outbreak response strategies worldwide. These strategies seek to ensure early and timely identification of cases, their effective isolation, and rapid contact tracing to break the chain of transmission [11]. For this purpose, the WHO African Regional Office has prioritized use of Go.Data among existing WHO electronic data collection platforms in the COVID-19 outbreak context. This tool has been recently developed by the WHO in collaboration with partners in the Global Outbreak Alert and Response Network [12].

Go.Data is an outbreak investigation tool designed for flexibility in field data collection during public health emergencies. This tool provides functionality for case investigation, contact tracing, and visualization of transmission chains. It facilitates data collection about an outbreak of an infectious disease, including cases associated with that outbreak, events at which transmission of the disease may have occurred, contacts that have been at risk of infection through exposure to a case or event, and contact tracing to monitor their health following an exposure [12,13].

This electronic platform assists responders to choose the right interventions to stop the disease from spreading and to work smarter [14]. The key difference between Go.Data and other existing WHO electronic systems in the WHO African Region is that the Go.Data tool has been designed primarily to improve contact tracing activities to break disease transmission. It also has a functionality for visualization of transmission hierarchies. This is in contrast to other existing WHO electronic surveillance systems that have the primary focus to support EWARS functions as part of routine Integrated Disease Surveillance and Response (IDSR) activities [8,9,15].

The complete rollout of an electronic field data collection platform ranges from a couple of weeks to a month for planning, deployment, training, and support including regular technical advice in the WHO African Region. Potentially, this may leave a critical gap between the declaration of the onset of a COVID-19 outbreak and the start of data collection that is in line with operationalization delays reported with existing electronic platforms for EWARS [8,16-18]. Few earlier studies have highlighted a need to use an interim and rapidly deployable solution that could bridge the gap between outbreak onset and full implementation of electronic data systems. Studies also suggest considering poor technical capacity and issues with access and resources when implementing such interventions in resource-poor settings [8,17].

This paper discusses the process our team undertook to research and develop a tool that would bridge the data collection gap between the onset of an outbreak and the start of data collection using a prioritized electronic data collection platform (PEDCP). We developed the COVID-19 Data Summarization and Visualization (DSV) tool for this purpose. Currently, this DSV tool is used in the 18 countries of the WHO African Region to support the current COVID-19 outbreak response. Additionally, we highlight preliminary findings and lessons learned from the DSV tool deployment in the 18 countries in the WHO African Region.

## Methods

### Review

To inform prioritized development of the COVID-19 DSV tool and better understand functions of existing tools against targeted needs, we reviewed the use of existing WHO electronic platforms and software for EWARS, case-based surveillance, contact tracing, and other eIDSR-related surveillance activities during various outbreaks and health emergencies in the WHO African Region. These included EWARS-in-a-Box, eDEWS, and Go.Data. The purpose of the review was also to identify any existing minimum data system standards aligned with COVID-19 regional and global surveillance guidelines and protocols; to prioritize information needs for effective COVID-19 outbreak response, planning, and decision making; and to inform the design of the interim DSV solution to facilitate smooth transitioning to implementation and full deployment of a PEDCP [19,20].

The review was conducted through an online literature search for technical guidance documents and published data in PubMed/MEDLINE (Medical Literature Analysis and Retrieval System Online), and Google Scholar databases including the WHO library database; directly approaching WHO teams, by phone call, by emails, or in person, involved in designing and implementing these platforms in the WHO African Region; and informal focus group discussions with WHO operational staff, including members of field teams, managers, and leaders involved in field data collection and contact tracing during health emergencies such as Ebola virus disease in the Democratic Republic of the Congo and West Africa, and other outbreaks in the WHO African Region.

### DSV Tool Design

As a result of our review, we identified the following seven key considerations essential in the development of the tool.

1. Inclusion of WHO standard data needs and priorities for the COVID-19 response in the African Region
2. Challenges around field data collection, management, analysis, and visualization during the COVID-19 outbreak in member states
3. Reporting requirements under the WHO International Health Regulations (2005), namely data flow from the field to country to regional office
4. Facilitate smooth deployment of robust data collection and contact tracing solution for case-based reporting
5. Challenges around frequent staff turnover and training needs
6. Lack of basic infrastructure with key technical, political, and financial considerations
7. Ease of use, sustainability, and local ownership

We also concluded that the tool must be specifically designed for interim use, rapidly deployable, cost effective, and time efficient.

**Figure 1:**
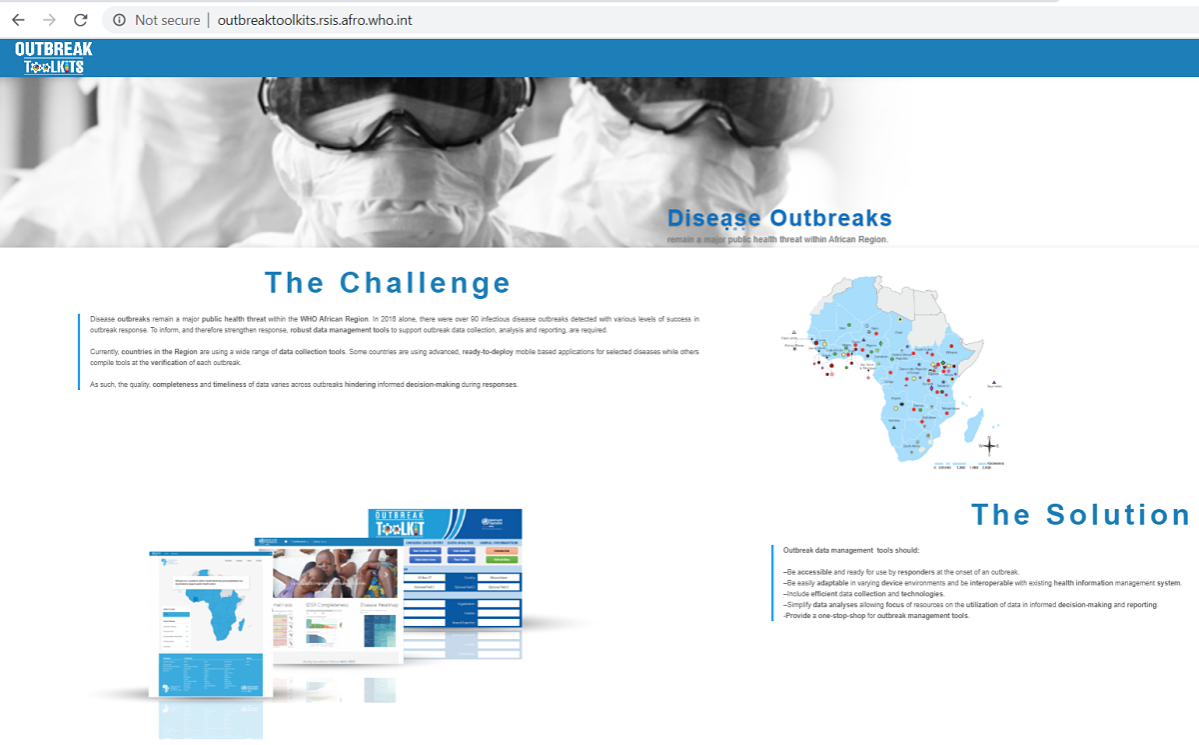
Screenshot of publicly available web portal of the World Health Organization Regional Office for Africa Toolkit Project.

**Figure 2:**
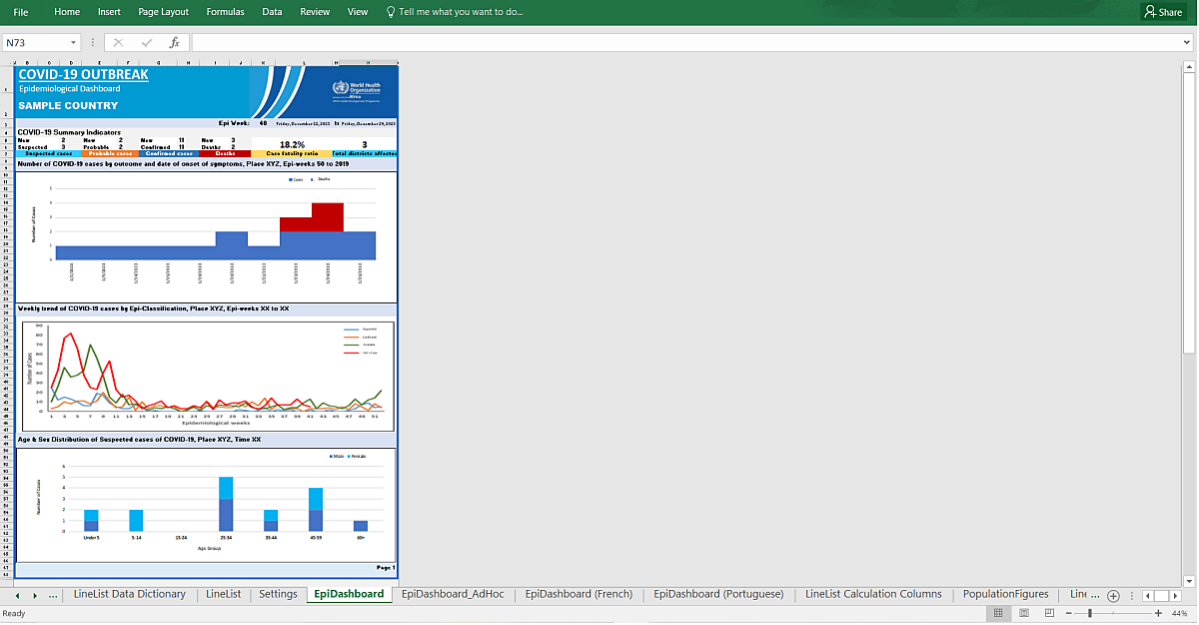
Screenshot of publicly available Data Summarization and Visualization tool showing automated printable data visualization.

The DSV tool was developed as part of the WHO Regional Office for Africa (AFRO) initiative “Outbreak Toolkits,” available publicly at the WHO outbreak toolkit web portal [21], that was adapted later at the WHO Global level for replication in the global perspective in other WHO regions across the world [22,23] (Figures 1 and 2).

## Results

### Development

We concluded based on our review that the DSV tool must be specifically designed for interim use, rapidly deployable, cost effective, and time efficient. The DSV tool was developed in line with the WHO Global COVID-19 Surveillance Guidelines and Protocols and adapted to the WHO African Region requirements. It assists member states in the collection, reporting, analysis, and interpretation of data *immediately* upon onset of any COVID-19 outbreak. The tool is simple, customizable, adaptable, and easy to implement for interim use.

We developed the DSV tool using Excel (Microsoft Corporation). This tool uses preformatted pivot tables with automation that is simple to use, requiring basic Excel skills at the end-user level. In phase two of the DSV tool deployment, we built automation processes to support export and integration of multiple COVID-19 data files for sharing by member states and to be merged into one central COVID-19 database for use at the WHO regional level. This DSV tool is located on a publicly available web portal for the COVID-19 WHO AFRO Outbreak Toolkit [13].

### Description of DSV Tool Modules

The DSV tool consists of modules for data collection, automated data management and analytics, and visualization. A brief description of each module follows.

#### Data Collection Module

We created the data collection tool using an Excel spreadsheet in the line listing format. We also created optional XLSForms for use in KoBoCollect, ODK, or other similar platforms for settings with prior XLSForm user experience and existing resources to use such platforms immediately. This allows flexibility for users either to enter data directly into formatted Excel worksheets with validation checks and conditional rules or to use XLSForms with recommended ODK or KoBoCollect platforms for data entry using electronic devices as an option.

#### Automated Data Management and Analytics Module

We created preformatted pivot tables with functionality to automatically extract the significance from a large, detailed data set; summarize it into tables; and then use canned pivot analysis for rapid multidimensional and meaningful high-level analysis of data. We kept flexibility to allow an ad hoc type of data handling and analysis as needed.

#### One-Click Spreadsheet Visualization: COVID-19 Dashboard

To help with data analysis and interpretation, we created preformatted single-click visualizations and locked layouts of the COVID-19 dashboard to facilitate printing of the dashboard in PDF format and quickly share initial and high-level summary with health emergency managers, leadership, and other stakeholders in the WHO African Region.

#### Multilingual Support

This includes templates in three languages English, French, and Portuguese to address the priority languages used in the WHO African Region.

### Using the DSV Tool as an Immediate Response for COVID-19 Outbreaks

It is specifically recommended that the DSV tool is immediately deployed upon confirmation of the first COVID-19 case within the African Region. Rapid deployment will support the rollout of a prioritized electronic tool, which is currently facing delays due to country-applied public health and social measures including movement restrictions and geographical area quarantine in settings lacking basic infrastructure and necessary resources. To facilitate immediate reporting with the DSV tool, we have also developed preconfigured XLSForms in multiple languages to be used with KoBoCollect, ODK, or any other platform that supports XLSForm configurations to facilitate real-time collection of data from the field using electronic devices and kept as an optional feature for use.

**Figure 3:**
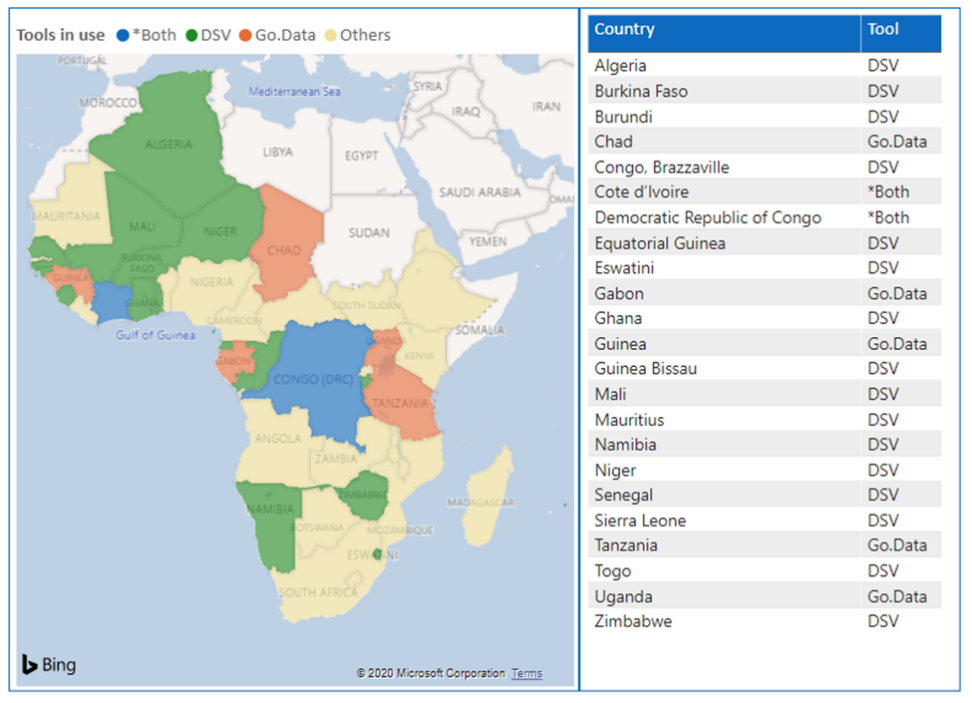
Map showing the status of COVID-19 data collection tools deployed by the World Health Organization African Regional Office in the member states, as of April 2020. DSV: Data Summarization and Visualization.

The main objective of developing the DSV tool was to provide an interim solution for immediate deployment during the COVID-19 outbreak response for field data collection, contact tracing follow-up, and generating epidemiological information for decision makers in a timely manner. As shown in Figure 3, as of May 10, 2020, the DSV tool has been deployed in 18 member states in the WHO African Region and has been shared with other member states as part of a readiness and preparedness package. The interim use of the DSV tool is recommended to avoid delays in settings where technical infrastructure and constraints on resources remain major barriers to launching any electronic data collection platform for COVID-19 case-based surveillance.

### Automation for COVID-19 Data Management for Decision Making

**Figure 4:**
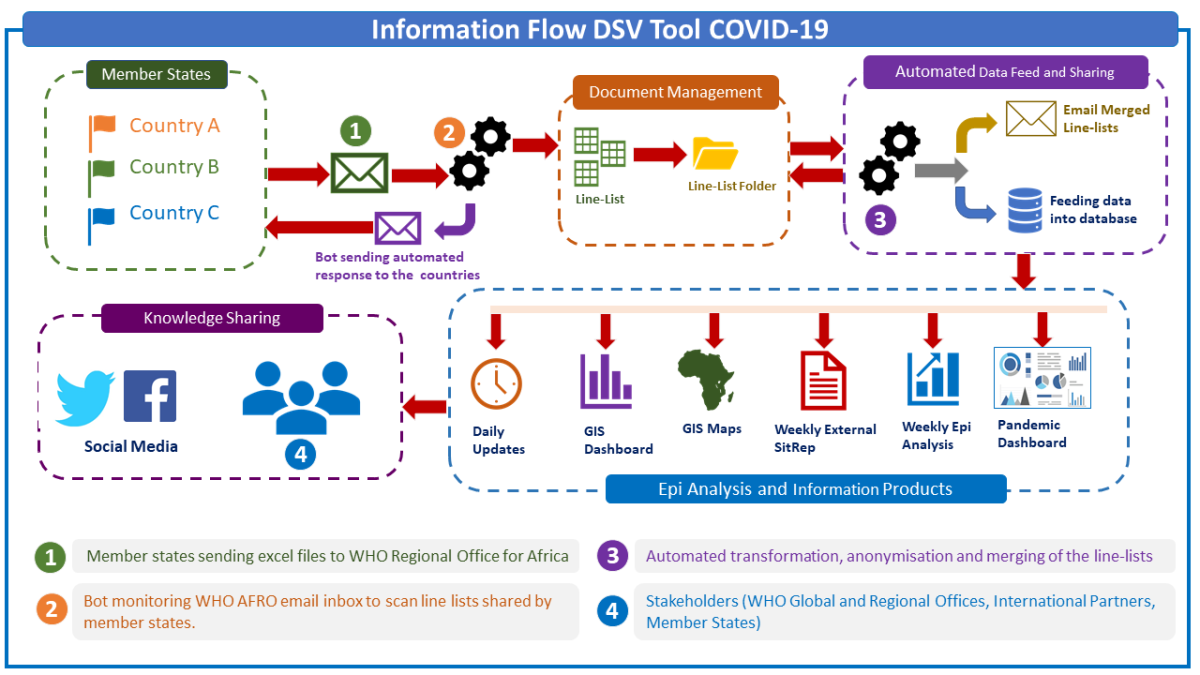
Automation workflow showing management of COVID-19 data in the WHO African Region. AFRO: Regional Office of Africa; DSV: Data Summarization and Visualization; GIS: geographic information system; WHO: World Health Organization.

Figure 4 shows how data extracted using the DSV tool flows from member states to the WHO AFRO office. At the WHO AFRO office, all COVID-19 data in Excel files are received via email after the data validation process at the WHO country offices are completed and stored using an automated document management platform. At the WHO AFRO office, the COVID-19 data files are merged, compiled, and shared with the data analytics teams. The data are then translated into information using R analysis for evaluation, synthesized into evidence, and stored in an online-integrated data warehouse with a front-end web portal and COVID-19 dashboard. At this stage, synthesized information on the COVID-19 outbreak is translated into knowledge and shared with stakeholders (WHO global and regional offices, international partners, and member states) using authoritative products such as AFRO COVID-19 daily updates, weekly situation reports, weekly epidemiological updates, geographic information system maps, and COVID-19 pandemic dashboards.

### Transitioning Timeline

For a PEDCP, the planning, procurement, deployment, training, and support activities required resulted in a delayed rollout during the recent deployments in the WHO African Region. The planning phase usually includes predeployment reviews, requirement analysis, procurements, testing, and software configurations. This phase is followed by a software deployment, which includes infrastructure provisioning, software installation support, training of trainers, and customization and adaptation of data collection forms, after which the system goes live. The last phase is end-user training and maintenance with sustained software support to start data collection and maintain the system.

**Figure 5:**
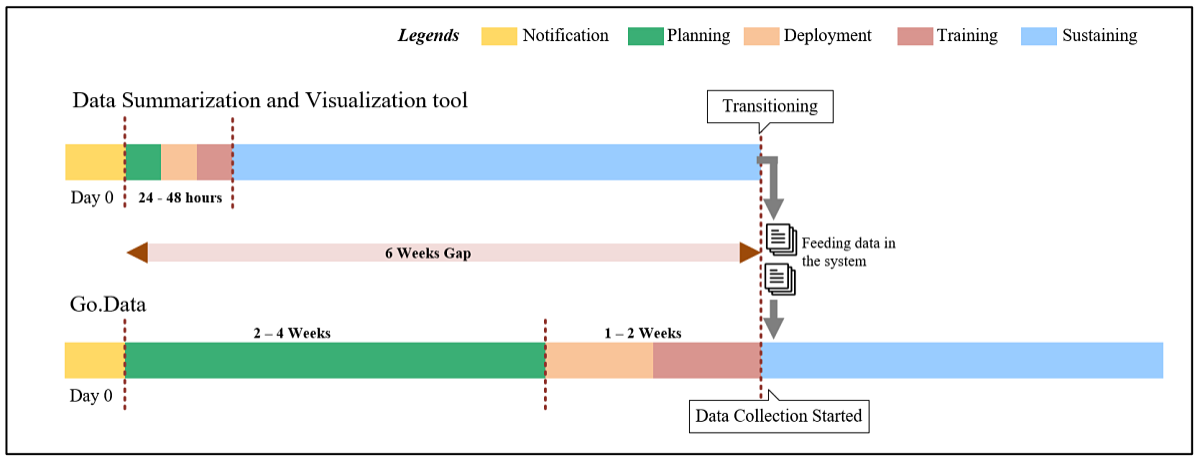
Timeline showing transitioning of Data Summarization and Visualization tool to Go.Data prioritized by the World Health Organization (WHO) Regional Office of Africa for COVID-19 field data collection in the WHO African Region.

For the DSV tool, the simple preformatted Excel tool was shared with all member states in the WHO African Region as part of a readiness and preparedness package for the COVID-19 pandemic. The DSV tool comes with a quick user guide and takes 24-48 hours to customize for any additional requirements, with or without technical support from the WHO AFRO, and it is available for immediate data entry and visualization. The preformatted built-in automated analytics and visualization modules generate tables and charts in a PDF printable format. The deployment timeline comparison shows that the major benefit of the DSV tool is that it shortens tool deployment time and can be used to bridge the gap between data collection and contact tracing between the time of outbreak onset and the complete rollout of a robust electronic data collection software (Figure 5).

## Discussion

### Principal Findings

The DSV tool is not a replacement for a robust electronic data collection platform. However, this tool provides outbreak investigation and response teams with a means to start collection of COVID-19 case, laboratory, hospitalization, and contacts data immediately upon confirmation of a COVID-19 outbreak. In addition, it generates analytical data and visualizations in a timely manner using an automated process that shows the COVID-19 outbreak situation for emergency health managers and decision makers. As an Excel-based tool, it is specifically recommended as an interim solution for short-term outbreak needs and not for use in protracted outbreaks or emergencies. The Go.Data tool has been deployed in some member states of the WHO African Region, where it took several weeks for implementation and complete rollout since technical support was not possible on the ground due to country-applied public health and social measures including movement restrictions and geographical area quarantine. The WHO AFRO is working closely with partners on a range of similar activities and finding ways to work around limitations imposed by travel restrictions and site presence.

The deployment of a PEDCP needs time for preparations, planning (requirement analysis, budget, and action plan), procurements, customizations, configurations, training, and support. This increases the inevitable time delay between onset of COVID-19 outbreak and complete rollout, especially in unprepared settings where electronic tool deployment has not been considered as part of a WHO AFRO country readiness and preparedness plan for a COVID-19 outbreak. This paper presents preliminary findings of this work, and an evaluation is planned, under the framework for the health emergency information management in the WHO African Region, to assess how well this intervention achieved its goals (simplicity, cost effectiveness, time efficiency, etc), what worked well, what did not work well, and how to improve the effectiveness of operations.

As part of preparedness and response, the WHO AFRO Health Information Management and Risk Assessment Program developed the DSV tool as an interim solution to support the immediate collection of case and contact data during the initial phase of a COVID-19 outbreak response. The main purpose of this tool was to bridge the critical time delay between a COVID-19 outbreak onset and PEDCP deployment for field data collection and contact tracing. To date, the DSV tool has been successfully implemented in 18 member states as an interim approach in parallel to planning for the Go.Data rollout as a prioritized electronic platform.

There are three main official languages (English, French, and Portuguese) spoken in the WHO African Region where the DSV tool was deployed. We developed built-in multilingual support in the DSV tool by including templates in these three official languages as a standard approach for the region. We have kept flexibility in the tool to add more language templates so other spoken local languages can be easily configured in the tool and facilitate immediate availability of translated text in the tool. This functionality has been reported to be useful in overcoming the language barrier with implementation in the WHO African Region.

A workflow has been developed using automated tools at the WHO regional office to support merging of multiple COVID-19 data files coming from the member states into one master data set. Information management teams then use this master data set to perform analysis on the COVID-19 outbreak situation in the WHO African Region and produce authoritative information products. Additionally, this data is further integrated with other relevant information coming from other health pillars and evaluated in terms of broader stakeholders’ needs and issues confronting the health emergency.

In the past few years, the WHO has developed and deployed multiple electronic data systems to support EWARS functions and routine disease surveillance, and these tools have been found to be effective and efficient informatics solutions in those settings. However, limitations have been reported in addressing some specific case-based surveillance and contact tracing needs [8,16]. We learned during the literature review and informed discussions that the EWARS-in-a-Box and eDEWS platforms were developed by the WHO with the objective of early detection and response to disease outbreaks during health emergencies. These platforms produced encouraging results with scaling up to other countries for EWARS and IDSR programs. However, both systems were not designed to support collection and management of complex contact tracing data to break the community disease transmission [8,9,15,18]. On the contrary, Go.Data has been designed to address this critical gap along with a focus on collecting and managing complex contact tracing data efficiently with visualization tools to support response efforts more effectively during outbreaks and emergencies [12].

Finally, the DSV tool provides an innovative and low cost simple analytical and visualization interim solution for data collection and management. The cost was low because this project was developed and implemented without dedicated funds and only used existing infrastructure and resources at the WHO regional office, the WHO country offices, and health authorities in the member states. The DSV tool successfully bridges the critical gap between COVID-19 outbreak onset and PEDCP operationalization to avoid delays in getting critical COVID-19 data in a timely manner during this period, facilitate timely access to validated COVID-19 data, and enable translation of data into actionable information to support swift public health response and decision making both at the country level and at the WHO African regional office. The DSV tool is easily and immediately deployable in practice using existing infrastructure and resources, and has been developed using a time efficient Excel pivot table technique that requires basic Excel skills at the end-user level, and tool standardization using the WHO regional and global guidelines and protocols makes it usable across all member states in the WHO African Region. We avoided the use of Visual Basic for Applications (VBA) macros to address possible tool performance issues and made it compatible with multiple versions ranging from Excel 2013 to Excel for Office 365, including compatibility with multiple operating systems. The built-in visualization module generates automated epidemiological reports on a timely and ad hoc basis, an important public health informatics approach to perform well during the COVID-19 emergency. Building local capacity to use the DSV tool for complete data analysis, visualization, and reporting is easy using remote webinar sessions where basic Excel knowledge is considered essential for health staff participation. The DSV approach also improved timeliness of information sharing on epidemiological trends and feedback to field teams and key stakeholders involved in the outbreak response.

### Lessons Learned

Drawing on the WHO AFRO team’s experience in planning and conducting DSV tool deployment activities in 18 member states, we describe the following critical lessons learned and offer an opportunity to learn and apply these lessons to improve future similar public health informatics initiatives, including (but not limited to) COVID-19, at any outbreak or similar humanitarian setting.

The deployment of the DSV tool was smooth since most of the data managers in the WHO African Region are familiar with Excel-based tools and quickly adapted the DSV tool using technical guidance guidelines provided for the local COVID-19 outbreak context. The main concern shared by the data management team was that a reasonable number of variables be collected by the tool in considering the impact on staff workload and that the more than 50 variables proposed in the COVID-19 surveillance guidelines presented a challenge. Another challenge was to identify minimum standard variables in the WHO African regional context from the list of 87 variables recommended for COVID-19 surveillance in the WHO technical guidance document [24]. The tool was then designed to ease the workload for data entry, where we identified 22 minimum standard variables as required inputs and kept other variables as optional, based on feedback provided during the deployment in member states.

During the planning phase, our technical staff experienced in-field data collection in the WHO African Region suggested to limit end user exposure with VBA-enabled functions to avoid potential tool performance issues since, based on experience, it is not reliable. However, use of macro-enabled workbooks can give better results when adapted in small scale only. Another notable observation to highlight here is that, when considering bulk data entry, users expressed a preference for spreadsheet applications over form-based data entry applications. Spreadsheets are quick and flexible to establish and adapt, and they allow faster data entry through the use of copy and paste, and drag and drop functionalities to facilitate data entry and manipulation. Despite offering benefits such as more robust data validation, form-based data entry applications require more technical skills to establish and typically only allow working on a single observation at a time and thus take considerably longer when manipulating large volumes of data.

A common technical issue with the DSV tool was experienced by some member states. It was reported that there were problems handling date systems (1904 and 1900) compatibility between operating systems Macintosh, iOS, and Windows. This compatibility issue did not affect data entry processes using the DSV tool but required careful processing when compiling workbooks generated from different operating systems. To resolve this issue permanently, we developed a VBA-based plug-in and installed it in the DSV tool as an update.

The adoption of the DSV tool was smoother in countries where the WHO country office data management teams had stronger relationships with their Ministry of Health counterparts. Building on existing working relationships enabled faster collaboration and decision making on the selection of data collection tools and establishment of data collection processes and reporting channels. Furthermore, member states that are stronger in surveillance have tended to require less support for establishing data collection platforms and are better able to leverage and integrate existing investments in IDSR toward outbreak response. Therefore, there is a need for strengthening data management capacity in member states with weak surveillance mechanisms. The team observed limitations within some countries for establishing data collection systems, defining and documenting data management processes, integrating data from multiple sources, and managing line lists. Some of these challenges manifested from limited expertise, lack of well-defined standard operating procedures for data management in the context of outbreaks, lack of clearly defined roles and responsibilities within and across teams, and slow activation and repurposing of existing staff onto the COVID-19 response.

### Limitations

Two slightly different naming approaches have been used in the past for the same concept of the WHO’s EWARS, a component of an integrated disease surveillance during various health emergencies across the world. These names are EWARS, Disease Early Warning System, and Early Warning, Alert and Response Network (EWARN). To keep consistency across the paper, we have used the term “EWARS” from the most recent naming convention in the WHO’s Emergency Reforms Framework, but the concept of EWARN is the same as EWARS and should not be confused when referring to other earlier papers.

### Conclusion

In conclusion, we developed an innovative tool for time efficient COVID-19 data collection, management, summarization, and visualization for immediate deployment in COVID-19 outbreak settings of member states in the WHO African Region. The automation process was introduced to facilitate timely knowledge sharing with response teams and decision makers, who rely on timely and accurate information for evidence-based decision making. The approach and processes used in, and the lessons learned from, this paper are generalizable to other health emergencies and need to be considered as an interim solution for rapid deployment and immediate field data collection needs while deployment of an electronic platform or software like Go.Data, EWARS-in-a-Box, or eDEWS is planned for the next health emergency.

## Abbreviations

AFRO: Regional Office for Africa
DSV: Data Summarization and Visualization
eDEWS: electronic Disease Early Warning System
eIDSR: electronic Integrated Disease Surveillance and Response
EWARN: Early Warning, Alert and Response Network
EWARS: Early Warning, Alert and Response System
IDSR: Integrated Disease Surveillance and Response
MEDLINE: Medical Literature Analysis and Retrieval System Online
PEDCP: prioritized electronic data collection platform
VBA: Visual Basic for Applications
WHO: World Health Organization
